# *Salvia libanotica* improves glycemia and serum lipid profile in rats fed a high fat diet

**DOI:** 10.1186/s12906-015-0917-8

**Published:** 2015-10-23

**Authors:** Maya Bassil, Costantine F Daher, Mohammad Mroueh, Nadine Zeeni

**Affiliations:** Department of Natural Sciences, School of Arts and Sciences, Lebanese American University, Beirut, Lebanon; School of Pharmacy, Lebanese American University, Beirut, Lebanon

**Keywords:** *Salvia libanotica*, Glycemia, Blood glucose, Blood lipid, Blood cholesterol

## Abstract

**Background:**

*Salvia libanotica* (*S. Libanotica*) is a commonly used herb in folk medicine in Lebanon and the Middle East. The present study aimed to assess the scientific basis for the therapeutic use of *S. libanotica* in glycemia and to evaluate its effects on lipemia and abdominal fat.

**Methods:**

Animals were fed a high-fat diet and allocated into a control and three experimental groups (GI, GII and GIII) receiving incremental doses of the plant water extract in drinking water (50, 150 and 450 mg/Kg body weight respectively) for six weeks.

**Results:**

The intake of *S. libanotica* extract was associated with a significant decrease in fasting serum glucose (102.9 ± 10.8 in GII and 87.5 ± 6.4 in GIII vs. 152.1 ± 7.9 mg/dl in controls) and a two fold increase in fasting serum insulin (GIII) and liver glycogen content (GII and GIII). Group III also had better glucose tolerance following intraperitoneal glucose challenges. Additionally, the plant extract intake produced a significant improvement in serum HDL (34.4 ± 2.4 in GIII vs. 27.2 ± 1.9 mg/dl in controls) and HDL/LDL cholesterol ratio (2.79 ± 0.32 in GII and 3.02 ± 0.31 in GIII vs. 1.74 ± 0.18 in controls), as well as a decrease in abdominal fat.

**Conclusion:**

The current study is the first to demonstrate that the chronic intake of *S. libanotica* infusion helps in the prevention of high fat-induced hyperglycemia and dyslipidemia. This supports the plant use as a remedy for the prevention of type 2 diabetes and cardiovascular diseases.

## Background

Diabetes and cardiovascular diseases are established as serious public health problems around the globe and are among the top five causes of death in most Western and Arab countries [1, 2]. Insulin resistance, hyperglycemia and dyslipidemia are common metabolic abnormalities that could potentially lead to type 2 diabetes and cardiovascular diseases [3]. A very palatable diet that is widely available nowadays is the high fat diet, rich in saturated fats, which is known to contribute to insulin resistance especially when it induces abdominal obesity [4]. This is characterized by impaired insulin-stimulated glucose uptake by peripheral tissues and subsequent hyperglycemia, in addition to abnormalities in hepatic insulin signaling leading to elevated blood lipids and gluconeogenesis and lower glycogen and HDL levels [5]. While pharmacological treatments are widely prescribed to manage these abnormalities, most are costly and have adverse side effects [6]. Thus, many patients and healthcare professionals are seeking alternative treatments by using herbal remedies due to their potential effectiveness and limited cost and toxicity [7].

*Salvia libanotica* (also named *S. Fruiticosa* and *S. triloba*) of the “Lamiaceae” family is an aromatic medicinal plant commonly known as East Mediterranean sage or Lebanese sage [8]. In Lebanon, Syria and Jordan, the plant is commercially available and widely used by the elderly and folk medicine practitioners [9]. Its leaves are usually used as infusion for the relief of headaches, stomachaches, abdominal pain, hyperglycemia and many other ailments [10–13].

In Lebanese folk medicine, S*. libanotica* is widely used for its anti-diabetic properties in spite of the scarcity of scientific evidence of its efficacy in the literature [9]. To our knowledge, only one study described the hypoglycemic effects *S. libanotica* when using a 10 % infusion of the plant leaves at an oral dose of 250 mg of dry leaves/kg body weight in rabbits [14]. The proposed mechanisms that were hypothesized by the authors included a role of the plant in carbohydrate intestinal absorption and/or modification of hepatic metabolic processes, since no effect was observed on plasma insulin [14]. On the other hand, many studies have established the hypoglycemic effect of leaves of the closely related to *Salvia officinalis* (*S. officinalis)* species. Indeed, *S. officinalis* methanolic extract was shown to significantly decrease serum glucose in diabetic but not healthy rats, with no effect on pancreatic insulin release [15, 16]. Another study showed that water-ethanol extract of *S. officinalis* reduced blood glucose in normoglycemic and mildly alloxan-diabetic, but not in severely alloxan-diabetic mice [17]. Additionally, replacing water with *S. officinalis* infusion for 14 days resulted in lower fasting plasma glucose with no effect on glucose clearance in normal mice [18].

The effects of *S. libanotica* on blood lipemia have not previously been studied. Yet, *S. officinalis* was shown to have potential cardio-protective effect by increasing blood HDL cholesterol levels and decreasing total cholesterol, triglyceride and LDL cholesterol levels in hyperlipidemic patients [19]. While the health benefits of S. *officinalis* plant extract are relatively well established, the effects of *S. libanotica* on glycemia and lipidemia have not been fully elucidated. Moreover, most studies on *S. libanotica* are short term studies [12], and have been conducted on its essential oil rather than on water extract [20–22]. In order to simulate human consumption and folk medicine protocols which rely on herbal infusions [14, 20–22], the present study was carried out to assess the effect of chronic intake (6 weeks) of *S. libanotica* water extract on glycemia and lipemia and explore the associated mechanisms of action in healthy rats fed a high-fat diet.

## Methods

### Plant material

*S. libanotica* plant was collected from different areas throughout Lebanon between July and September 2013. The plant was identified according to the characteristics described in “Handbook of Medicinal Herbs” book [23]. Also, a voucher specimen of the plant material used has been deposited at the herbarium of the Department of Natural Sciences, Lebanese American University (reference number: SL-2-2013). Fresh plant material was collected from wild bushes; leaves were separated and dried in the shade at room temperature. The dried leaves (130 g) were then chopped into small pieces and soaked in 2 L pre-boiled water for 30 min for extraction. The water extract was filtered and subjected to freeze drying yielding 8.3 % w/w of dry material that was stored in refrigerated amber glass containers. This procedure was repeated weekly throughout the study.

### Animals

Adults Spargue-Dawley rats (Lebanese American University Stock) weighing 180–240 g were housed in a temperature and humidity-controlled room under a 12:12 light/dark cycle (lights on at 0800 h). Rats were randomly allocated into four weight-matched groups of ten rats each and were studied for 6 weeks. Animals were fed *ad libitum* a standard chow diet to which 5 % of coconut oil was added in order to increase the diet’s atherogenicity (Table 1) [24]. The control group was given water, and the other groups were given three different doses of *S. libanotica* in drinking water (GI: 50 mg/Kg body weight; GII: 150 mg/Kg body weight; GIII: 450 mg/Kg body weight). All experimental protocols were approved by the Animal Ethical subcommittee of the Lebanese American University, which complies with Guide for the Care and Use of Laboratory Animals [25].

**Table 1:** Nutrient composition of the high fat diet compared to standard high carbohydrate (HC) diet

	Standard HC^a^	High Fat^b^
Protein (% wt)	19	18.1
Carbohydrates (% wt)	65.3	62.2
Sugars	9.2	8.8
Fat (% wt)	9.6	13.9
Fat breakdown		
Saturated fat	18 %	21 %
Mono-unsaturated fat	29 %	28 %
Poly-unsaturated fat	47 %	45 %
Fiber (% wt)	4.3	4.1
Metabolizable energy (kJ/g)	17.7	18.7
Energy (Protein)	18 %	16 %
Energy (Carbohydrate)	62 %	56 %
Energy (Fat)	20 %	28 %

^a^Laboratory rodent starter diet no. 1, Hawa Chicken Co. (Safra, Lebanon)

^b^Diet derived from the standard HC diet enriched with coconut oil

### Glucose tolerance test

On days 17 and 36 of the experiment, an ipGTT (intraperitoneal glucose tolerance test using intraperitoneal injection of 300 g glucose/L in physiologic saline in a dose of 5.83 mL/Kg boy weight) was performed on rats that have been fasted for 3 h. After 45 min, blood was collected from the animals’ tail vein and assayed for glucose level.

### Metabolic profile

After 6 weeks, fasted rats (18 h) were anesthetized and sacrificed after 18 h. Serum samples were isolated from 4 ml of venous blood which were drawn from the inferior vena cava and stored at −80 °C for subsequent analysis. Serum glucose, lipids (total cholesterol, HDL cholesterol, triglycerides) and liver enzyme activities of aspartate transaminase (AST), alanine transaminase (ALT) and alkaline phosphatase (ALP) were determined using the relevant Spinreact kits (Spinreact, Spain). LDL cholesterol was calculated using the Friedwald equation (LDL = total cholesterol – HDL – (triglycerides/5)) [26]. Serum insulin was determined using Rat-insulin ELISA kit (Merck Millipore,Germany). All serum samples were run in duplicate and analyzed within the same assay. In addition, following sacrifice, the intra-abdominal fat (epididymal, mesenteric and retroperitoneal) was removed from the animals, after which it was cleaned, blotted on a filter paper and weighed.

### Liver glycogen content

Rats’ livers were isolated, weighed, and then preserved at −45 °C until testing. Hepatic glycogen was assayed using 1 g of tissue according to the extraction method mentioned by Hassid and Abraham [27]. Briefly, 1 g of tissue was homogenized with 10 % trichloroacetic acid solution and glycogen was precipitated using 95 % ethanol. After diluting it with distilled water, glycogen was then determined colorimetrically by the anthrone method.

### In-vitro digestive enzymes inhibition assays

The effects of *S. libanotica* on the intestinal enzymes alpha-amylase and alpha-glucosidase were determined in-vitro [28, 29]. Solutions of plant extracts were prepared at concentrations of 10, 20, 40, 60, 80 and 100 mg/dl. Controls were prepared by replacing the plant extract with distilled water, and acarbose was used as the positive control.

### In-vitro alpha-amylase inhibition assay

For each concentration, 1 ml of the plant extract solution was mixed with 1 ml of 0.5 % starch solution and 1 ml of enzyme solution (0.5–1 unit/ml in 20 mM sodium phosphate buffer, pH:6.9), and the mixture was incubated at 25 °C for 3 min. Afterwards, 1 ml of color reagent was added. The latter was prepared by mixing 96 nM of 3,5 dinitrosalicylic acid (20 ml) with 5.31 M sodium potassium tartrate in 2 M sodium hydroxide (8 ml), all made up to 12 ml volume with deionized water. The mixture was placed in closed tubes in a water bath at 85 °C for 15 min. Tubes were then removed from water bath, cooled and diluted with 9 ml of distilled water and absorbance was read at 540 nm in a spectrophotometer.

$$ {\mathrm{I}}_{\alpha -\mathrm{amylase}}\% = 100\ \mathrm{x}\ \left(\varDelta {\mathrm{A}}_{\mathrm{Control}}-\varDelta {\mathrm{A}}_{\mathrm{Sample}}\right)\ /\ \left(\varDelta {\mathrm{A}}_{\mathrm{control}}\right) $$

Where *Δ*A_Control_ = A_test_ − A_Blank_ and *Δ*A_sample_ = A_test_ − A_Blank_

### In-vitro alpha-glucosidase inhibition assay

Alpha-glucosidase reaction mixture was prepared by adding 2.9 mM p-nitrophenyl- α-D-glucopyranoside (pNPG) to 0.25 mL of each plant extract concentrations and 0.6 U/ml α-glucosidase in sodium phosphate buffer (pH 6.9). The reaction mixture was incubated at 25 °C for 5 min and then the reaction was stopped by boiling for 2 min. Absorbance of the resulting p-nitrophenol (pNP) was determined at 405 nm using spectrophotometer.

$$ \%\ \mathrm{Glucosidase}\ \mathrm{inhibition} = 100\%-\%\kern0.5em \mathrm{a}\mathrm{ctivity}\ \mathrm{of}\ \mathrm{test}\ \mathrm{a}\mathrm{s}\ \mathrm{percentage}\ \mathrm{of}\ \mathrm{control} $$

$$ \%\ \mathrm{activity}\ \mathrm{of}\ \mathrm{test} = \mathrm{Corrected}\ \mathrm{A}405\ \mathrm{of}\ \mathrm{test}\ \mathrm{x}\ 100\%/\mathrm{A}405\ \mathrm{of}\ \mathrm{controls} $$

### Statistical analysis

Data are reported as Mean ± SEM. Results were analyzed by one-way analysis of variance (ANOVA). Significant main effect differences were tested using Tukey–Kramer’s post hoc test for multiple comparison. All data were analyzed with the statistical package SPSS 18 and statistical significance was defined as *p* < 0.05.

## Results and discussion

### Results

**Fig. 1:**
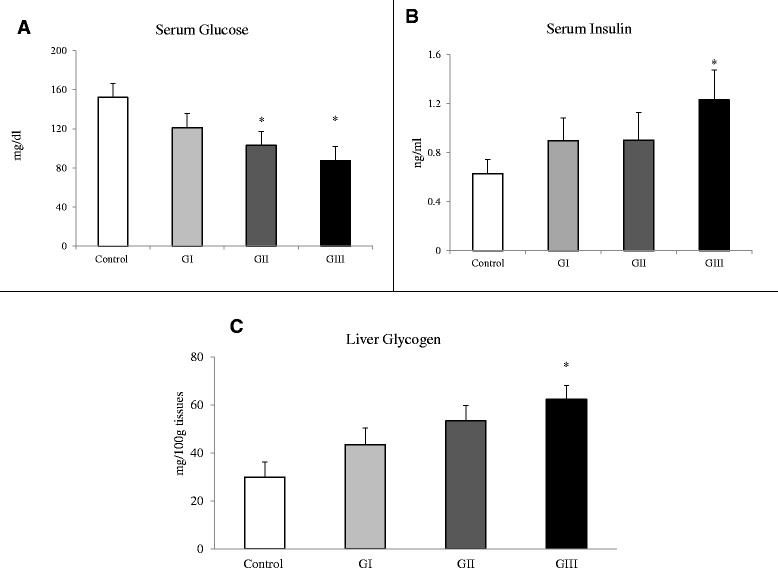
Fasting serum glucose and insulin, and liver glycogen content. Fasting serum glucose (**a**), insulin (**b**) and liver glycogen (**c**) after 6 weeks of *S. libanotica* intake. Data are presented as mean ± SEM, (*n* = 10). *corrected p-value ≤ 0.05 vs. control using ANOVA and Tukey’s post-hoc test

**Fig. 2:**
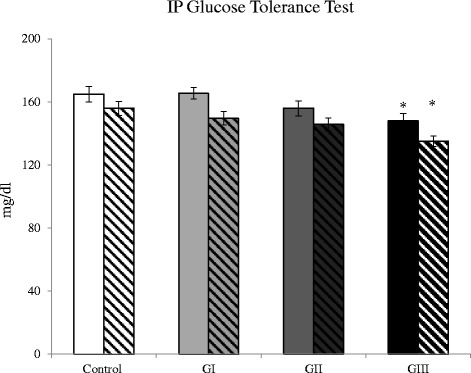
Glucose tolerance test. Serum glucose, 45 min after intraperitoneal injection of glucose solution (ipGTT) on days 17 (clear bars) and 36 (dashed bars) of the experiment. Data are presented as mean ± SEM, (*n* = 10). *corrected p-value ≤ 0.05 vs. control using ANOVA and Tukey’s post-hoc test

Chronic intake of *S. libanotica* extract showed a trend of weight loss in the majority of animals, with a significant decrease in abdominal fat percent in GIII (Table 2). Fasting serum glucose concentrations on day 42 of the study were lower in the treated groups compared to controls with the difference reaching statistical significance at the two highest doses used (Fig. 1a). Similarly, ipGTT on day 17 and day 36 of the study showed significantly (*p* < 0.05) lower venous blood glucose levels in GIII versus control group, 45 min after intrapertitoneal injection of glucose solution (Fig. 2). Additionally, the fasting serum insulin concentrations were higher in the treated groups compared to controls with significance (*p < 0.05*) reached at the highest dose (Fig. 1b). Furthermore, there was a dose dependent increase in liver glycogen with significance (*p* < 0.05) reached in GII and GIII (Fig. 1c).

**Table 2:** Body weight gain and abdominal fat after 6 weeks of *S. libanotica* intake

	Control	GI	GII	GIII
Body weight gain (g)	96.71 ± 5.31	76.33 ± 10.03	82.00 ± 5.84	71.20 ± 14.63
Abdominal fat (g)	2.96 ± 0.34	2.91 ± 0.37	2.61 ± 0.51	2.53 ± 0.41
Abdominal fat (% body weight)	0.78 ± 0.08	0.79 ± 0.10	0.74 ± 0.14	0.57 ± 0.06*

*Data are presented as mean ± SEM, (n = 10).* *corrected p-value ≤ 0.05 vs. control using ANOVA and Tukey’s post-hoc test

As for blood lipids, there was no significant difference in serum total cholesterol, LDL-cholesterol (LDL) and triglycerides among groups.

Conversely, plant treatment was associated with higher fasting serum HDL-cholesterol (HDL) and HDL/LDL ratio but the differences were only significant in GIII in the former and in GII and GIII in the latter compared to control group (Table 3).

**Table 3:** Fasting serum triglycerides, total cholesterol, LDL-cholesterol (LDL), HDL-cholesterol (HDL) and HDL/LDL ratio after 6 weeks of *S. libanotica* intake

	Control	GI	GII	GIII
Triglycerides (mg/dl)	37.9 ± 1.7	39.8 ± 2.1	35.4 ± 4.8	38.3 ± 4.2
Total Cholesterol (mg/dl)	49.8 ± 2.8	50.2 ± 3.3	51.4 ± 4.8	54.5 ± 3.1
LDL Cholesterol (mg/dl)	15.6 ± 2.3	15.1 ± 3.0	11.2 ± 3.3	11.4 ± 3.2
HDL Cholesterol (mg/dl)	27.2 ± 1.9	27.4 ± 2.5	31.2 ± 2.6	34.4 ± 2.4^*^
HDL/LDL ratio	1.74 ± 0.18	1.81 ± 0.25	2.79 ± 0.32^*^	3.02 ± 0.31^*^

*Data are presented as mean ± SEM, (n = 10).* *corrected p-value ≤ 0.05 vs. control using ANOVA and Tukey’s post-hoc test

Serum levels of AST and ALT in the different experimental groups were similar to those of the control. On the other hand, serum LDH levels in GII and GIII, but not GI, were significantly lower than that of the control group (Table 4). The dose dependent in vitro assay did not show any effect of the different doses of the plant extract, ranging from 10 to 100 mg/dl, on the intestinal enzymes *alpha-glucosidase* and *alpha-amylase*.

**Table 4:** Liver enzymes (AST, ALT and LDH) activities after 6 weeks of *S. libanotica* intake

	Control	GI	GII	GIII
AST	41.5 ± 1.84	37.4 ± 2.93	39.9 ± 3.23	35.10 ± 2.47
ALT	23.1 ± 1.60	21.8 ± 1.41	18.0 ± 1.34	20.4 ± 1.51
LDH	382 ± 15.5	360 ± 23.6	241 ± 27.3*	220 ± 25.3*

*Values are expressed as mean ± SEM (n = 10)*

*Data are presented as mean ± SEM, (n = 10).* *corrected p-value ≤ 0.05 vs. control using ANOVA and Tukey’s post-hoc test

### Discussion

The objective of the current study was to investigate the effect of chronic administration of *S. libanotica* water extract on glycemia, serum lipid profile, food intake, and abdominal fat of rats fed a high fat diet, as well as to explore potential mechanisms of action. The present work is the first to simulate human consumption of *S. libanotica* by mimicking its usual consumption as herbal infusion in the Middle East. Results showed a significant decrease in fasting blood glucose and an increase in serum insulin and liver glycogen. Moreover, treatment with plant extract was associated with improved lipid profiles characterized by higher HDL-cholesterol and HDL/LDL ratio, as well a decrease in abdominal fat percent.

Although not significant, chronic intake of the plant extract caused 15.2 to 26.4 % decrease in body weight gain in animals receiving the extract compared to control group. A similar decrease in abdominal fat ranging between 1.6 to 14.5 % of the animals was also observed. Moreover, calculation of the abdominal fat/body weight ratio showed a significant decrease in group III compared to control animals, suggesting a potential benefit of plant intake on abdominal fat deposition.

High fat diet administration for 6 weeks induced fasting hyperglycemia (152.1 ± 7.9 mg/dl) in the control group, while treatment with *S. libanotica* normalized fasting glucose at the two higher doses used (150 and 450 mg/Kg body weight). This appeared to be mediated by an insulinotropic effect of the plant water extract, which was demonstrated through the dose dependent increase in fasting serum insulin level. Therefore, we suggest that the plant treatment triggered more insulin secretion which might have increased glucose uptake by the tissues and thus lowered serum glycemia. This is in line with the higher liver glycogen content observed, which is also an indication of improved hepatic insulin sensitivity [30]. Such mechanism is supported by the *in-vitro* assessment of *alpha-glucosidase* and *alpha-amylase* activities, which did not reveal any inhibitory effect of *S. libanotica* water extract on these carbohydrate-digesting enzymes.

The plant hypoglycemic effect was also evident in the glucose tolerance test (ipGTT), whereby plant treatment at the dose of 450 mg/kg body weight (GIII) was associated with significantly lower glycemia, 45 min following an intrapertitoneal injection of glucose solution. This effect was even significant only after 17 days of plant treatment. This improved glucose tolerance could be the result of higher insulin response or increased insulin sensitivity, however none of these parameters was measured and thus further investigations are needed. Previous work by Perfumi et al. [14] showed lower blood glucose levels after an oral glucose load in normoglycemic and alloxan-induced hyperglycemic rabbits that were given a single oral dose of *S. fruticosa* infusion (at 250 mg dry leaves/kg body weight); however, no hypoglycemic effect was found after repeated administration of the infusion for seven days in normoglycemic rabbits. Moreover, the latter study did not find an effect of the plant extract on fasting or glucose-stimulated insulin levels. The discrepancy between the present results and those of Perfumi et al. [14] could be due to the differences in animal models used (rabbits vs. rats), mode of plant extract administration (single daily oral dose vs. drinking water) and study duration (7d vs. 42d). Moreover, based on the extract yield obtained from the current study, the dose used in Perfumi’s study would be about 21 mg water extract/kg bodyweight, which is substantially lower than the doses used in the present work.

The chronic intake of *S. libanotica* water extract significantly increased HDL cholesterol (group III) levels and HDL/LDL ratio (group II and III). The latter was found to be a strong marker of atherosclerosis compared to LDL and HDL alone [31], thereby suggesting a cardioprotective potential of the plant. To the best of our knowledge, this study is the first to demonstrate the dual hypolipidemic and hypoglycemic effect of the chronic intake of the plant extract. Both effects were not associated with toxicity since there was no elevation in serum activities of ALT and AST after 6 weeks of supplementation with the extract. Results also showed that LDH levels were significantly reduced in Group II and III, indicating the absence of pathological damage, as LDH is an enzyme that is found in almost all body cells and is released upon cell damage and destruction. Additionally, plant safety was further confirmed in our lab when a separate group of rats survived incremental oral doses of the plant extract reaching 8000 mg/Kg body weight.

## Conclusions

The present study demonstrated the first evidence of the hypoglycemic and hypolipidemic effect of the chronic intake of *S. Libanotica* water extract in healthy rats, and proved to be safe throughout the study. Therefore, the results suggest a promising role of *S. Libanotica* water extract in the prevention of chronic diseases such as diabetes, and cardiovascular diseases, with low cost and without compromising safety. Future studies are warranted to further explore mechanisms of action and to fractionate and purify the plant extract in order to identify the active ingredient(s) responsible for the observed beneficial effects.

## Abbreviations

ALP: Alkaline phosphatase
ALT: Alanine transaminase
AST: Aspartate transaminase
Groups I, II & III: GI, GII & GIII
HDL: High-density lipoprotein
ipGTT: Intraperitoneal glucose tolerance test
LDL: Low density lipoprotein
Salvia libanotica: S. libanotica
Salvia officinalis: S. officinalis
pNPG: p-nitrophenyl- α-D-glucopyranoside
ANOVA: One-way analysis of variance
SPSS: Statistical Package for the Social Sciences

## References

[1] World Health Organization (2014). World Health Statistics 2014.

[2] Abdul Rahim HF, Sibai A, Khader Y, Hwalla N, Fadhil I, Alsiyabi H, Mataria A, Mendis S, Mokdad A, Husseini AH (2014). Health in the Arab world A view from within two non-communicable diseases in the Arab world. Lancet.

[3] Karpe F, Dickmann RJ, Frayn NK (2011). Fatty acids, obesity, and insulin resistance, time for a reevaluation. Diabetes.

[4] Riccardi G, Giacco RA, Rivellese A (2004). Dietary fat, insulin sensitivity and the metabolic syndrome. Clin Nutr.

[5] Samuel VT, Shulman GI (2012). Mechanisms for insulin resistance, common threads and missing links. Cell.

[6] Inzucchi SE (2002). Oral antihyperglycemic therapy for type two diabetes scientific review. J Am Med Assoc.

[7] Saad B, Azaizeh H, Said O (2005). Tradition and perspectives of Arab herbal medicine: a review. Evid Based Complement Alternat Med.

[8] Mouterde P (1970). Nouvelle Flore du Liban et de la Syrie. Beyrouth (Lebanon).

[9] Gali-Muhtasib H, Hilan C, Khater C (2000). Traditional uses of Salvia Libanotica (east meditteranean sage) and the effects of its essential oils. J Ethnopharmacol.

[10] Hilan C, Khazzaka K, Sfeir R (1997). Antimicrobial effect of essential oil of Salvia libanotica (Sage). Br J Phytother.

[11] Todorov S, Philianos S, Petkov V, Harvala C, Zamfirova R, Olimpiou H (1984). Experimental pharmacologicalstudy of three species from genus Salvia. Acta Physiol Pharmacol Bulg.

[12] Dapkevicius A, Venskutonis R, Beek T, Linssen J (1998). Antioxidant activity of extracts obtained by different isola-tion procedures from some aromatic herbs grown in Lithuania. J Sci Food Agric.

[13] Schilcher H, Baerheim A, Svendsen JJ, Scheffer C (1985). Effects and side-effects of essential oils. Essential oils and aromatic plants.

[14] Perfumi M, Arnold N, Tacconi R (1991). Hypoglycemic activity of *Salvia Fructicosa* mill from Cyprus. J Ethnopharmacol.

[15] Eidi M, Eidi A, Zamanizadeh H (2005). Effects of Salvia Officinalis on serum glucose and insulin in healthy and Sterptozotocin-induced diabetic rats. J Ethnopharmacol.

[16] Eidi A, Eidi M, Darzi R (2009). Antidiabetic effect of Olea Europaea L. in normal and diabetic rats. Phytother Res.

[17] Alarcon-Aguilar FJ, Roman-Ramos R, Flores-Saenz JL, Aguirre-Garcia F (2002). Investigation on the hypoglycemic effects of extracts of four Mexican medicinal plants in normal and alloxan-diabetic mice. Phytother Res.

[18] Lima CF, Azevedo MF, Araujo R, Fernandes-Ferreira M, Pereira-Wilson C (2006). Metformin like effect of Salvia Officinalis (common sage): is it useful in diabetes prevention?. Br J Nutr.

[19] Kianbakht S, Abasi B, Perham M, Hashem Dabaghian F (2011). Antihyperlipidemic effects of Salvia officinalis L. leaf extract in patients with hyperlipidemia: a randomized double-blind placebo-controlled clinical trial. Phytother Res.

[20] Laude EA, Morice AH, Grattan TJ (1994). The antitussive effects of menthol, camphor and cineole in conscious guinea-pigs. Pulm Pharmacol.

[21] Pattnaik S, Subramanyam VR, Bapaji M, Kole CR (1997). Antibacterial and antifungal activity of aromatic constituents of essential oils. Microbios.

[22] Lachenmeier DW, Walch SG (2011). The choice of Thujone as drug for diabetes. Nat Prod Res.

[23] Duke JA, Bogenschtg-Godwin MJ (2002). Ducellier J.

[24] Daher CF, Koulajian KB, Haddad N, Baroody GM (2006). Effect of hydroxycut intake on fasted and postprandial lipemia in rats. J Toxicol Environ Health A.

[25] National research Council. Guide for the care and use of laboratory animals. Washington (DC), USA: The National Academies Press 2011.

[26] Friedewald WT, Levy RI, Fredrickson DS (1972). Estimation of the concentration of low-density lipoprotein cholesterol in plasma, without use of the preparative ultracentrifuge. Clin Chem.

[27] Hassid WF, Abraham S (1957). Chemical procedures for analysis of polysaccharide. Acad Press New York.

[28] Nickavar B, Abolhasani L, Izadpanah H (2008). α-amylase inhibitory activities of six Salvia species. Iran J Pharm Res.

[29] Matsui T, Yoshimoto C, Osajima K, Oki T, Osajima Y (1996). In vitro survey of α-lucosidase inhibitory food components. Biosci Biotechnol Biochem.

[30] Birkenfeld AL, Shulman GI (2014). Nonalcoholic fatty liver disease, hepatic insulin resistance, and type 2 diabetes. Hepatology.

[31] Enomoto M, Adachi H, Hirai Y, Fukami A, Satoh A, Otsuka M et al. LDL-C/HDL-C ratio predicts carotid intima-media thickness progression better than HDL-C or LDL-C alone. J Lipids 2011, doi:10.1155/2011/549137 10.1155/2011/549137 PMC3136137 21773051

